# Age and aging process alter the gut microbes

**DOI:** 10.18632/aging.205728

**Published:** 2024-04-08

**Authors:** Qu Zhanbo, Zhuang Jing, Han Shugao, Wu Yinhang, Chu Jian, Yu Xiang, Zhao Feimin, Liu Jian, Wu Xinyue, Wu Wei, Han Shuwen

**Affiliations:** 1 Fifth School of Clinical Medicine of Zhejiang Chinese Medical University (Huzhou Central Hospital), Huzhou 313000, Zhejiang, China; 2 Huzhou Central Hospital, Affiliated Central Hospital Huzhou University, Huzhou 313000, Zhejiang, China; 3 Key Laboratory of Multiomics Research and Clinical Transformation of Digestive Cancer, Huzhou 313000, Zhejiang, China; 4 The Second Hospital Affiliated to Zhejiang University School of Medicine, Hangzhou 310017, Zhejiang, China

**Keywords:** gut microbes, age, metagenomic sequencing, functional genes, bioinformatics analysis

## Abstract

Background: Gut microbes and age are both factors that influence the development of disease. The community structure of gut microbes is affected by age.

Objective: To plot time-dependent gut microbe profiles in individuals over 45 years old and explore the correlation between age and gut microbes.

Methods: Fecal samples were collected from 510 healthy individuals over 45 years old. Shannon index, Simpson index, Ace index, etc. were used to analyze the diversity of gut microbes. The beta diversity analysis, including non-metric multidimensional scaling (NMDS), was used to analyze community distribution. Linear discriminant analysis (LDA) and random forest (RF) algorithm were used to analyze the differences of gut microbes. Trend analysis was used to plot the abundances of characteristic gut microbes in different ages.

Results: The individuals aged 45-49 had the highest richness of gut bacteria. Fifteen characteristic gut microbes, including *Siphoviridae* and *Bifidobacterium breve*, were screened by RF algorithm. The abundance of *Ligiactobacillus* and *Microviridae* were higher in individuals older than 65 years. Moreover, the abundance of *Blautia_A massiliensis*, *Lubbockvirus* and *Enterocloster clostridioformis* decreased with age and the abundance of *Klebsiella variicola* and *Prevotell*a increased with age. The functional genes, such as human diseases and aging, were significantly different among different aged individuals.

Conclusions: The individuals in different ages have characteristic gut microbes. The changes in community structure of gut microbes may be related to age-induced diseases.

## INTRODUCTION

Gut microbes living in the human gut include bacteria, fungi, viruses, and archaea [1]. These microbes are numerous and diverse, constituting the gut microecosystem. They are important organs in human body with a wide range of functions [2]. First, they protect the gut from pathogenic bacteria and keep it healthy [3]. Second, gut microbes can promote food digestion and nutrient absorption, which contributes to human health [4]. In addition, gut microbes can also affect the function of the immune system [5], regulate the metabolism and endocrine of the human body [6], and have a certain impact on the health of the cardiovascular system and nervous system [7].

Gut microbes not only play an important physiological role in human body, their disorder and imbalance are also the cause of many diseases [8, 9]. These disorders are very likely to cause gastrointestinal problems, including inflammatory bowel disease [10] and colorectal cancer [11]. Moreover, it can also lead to a variety of underlying diseases, such as obesity [12], diabetes [13], heart disease [14], autoimmune diseases [15], and a host of other diseases.

Age is also a factor of many diseases. The function of human organs will change with age, thus affecting the body’s resistance ability and increasing the incidence of some diseases [16]. For example, hypertension [17], coronary heart disease [18] and diabetes [19] are all closely related to age. In addition, different diseases always occur at different ages. For instance, Alzheimer’s disease tends to affect individuals over 65 years old [20], Parkinson’s disease tends to affect individuals around 60 years old on average [21], and retinoblastoma tends to affect children under the age of 5 years old [22].

With the growth of age, human gut microecosystem will have certain changes [23]. The abundance, diversity, and composition of gut bacteria of the elderly are different from those of the young. In most cases, the gut bacteria of the elderly are more likely to be disturbed and have impaired function [24]. For example, the alpha diversity of the gut bifidobacterial microbiota decreased with age [25]. On the one hand, the body’s immune function varies with age, which leads to the imbalance of gut microbes. For example, older people generally have more harmful bacteria and an bacteria imbalance in their guts, as well as fewer beneficial bacteria [26]. On the other hand, aging brings changes in metabolic and endocrine functions, which also affect the community structure of gut microbes [27]. In short, age is closely related to gut microbes.

What’s more, the past studies always focused on bacteria in the gut, but recent research suggested that enteroviruses in the gut are also important [28]. Enteroviruses are more diverse than gut bacteria, including adenoviruses, noroviruses, coronaviruses and many other types [29]. Many studies have shown that enterovirus infections may be associated with conditions, such as inflammatory bowel disease [30], autoimmune diseases [31], and diabetes [32]. Enterovirus infection leads to a breakdown of the gut mucosal barrier, thus resulting in an inflammatory response [33]. Enteroviruses also affect the composition and function of gut bacteria, thus further affecting gut health [34]. In addition, enteroviruses have been associated with several autoimmune diseases such as systemic lupus erythematosus [35]. Therefore, the study of enteroviruses attracts more attention and may help us better understand the relationship between gut microbes and health [36].

Joint analysis of gut bacteria and enteroviruses provide a more comprehensive picture of gut microecology. Gut bacteria and enteroviruses are the main microbes in gut tract, and the development trend of gut bacteria and enterovirus is closely related [37]. Enteroviruses use certain gut bacteria as hosts to reproduce, and gut bacteria also influence the reproduction and spread of some enteroviruses, such as organoflaviridae and norovirus [38]. Therefore, when exploring the relationship between gut microbes and disease, combining gut bacteria and enteroviruses can show their interactions and influences in more detail. The host-virus-ratio (HVR) also shows a more complete picture of virus-bacterial correlation than possibly based on the combined virus and bacteria. After all, it takes more of the interactions involved into account.

This study explored the differences of gut microbes from the perspective of age, the age change trends of gut microbes were plotted, and the characteristic gut microbes of different ages were screened, which will lay the foundation for the study on the pathogenesis of age-related gut microbes. It provides a new direction for the mapping of standardized age maps of human intestinal microbes.

## RESULTS

### Descriptive analysis

**Figure 1:**
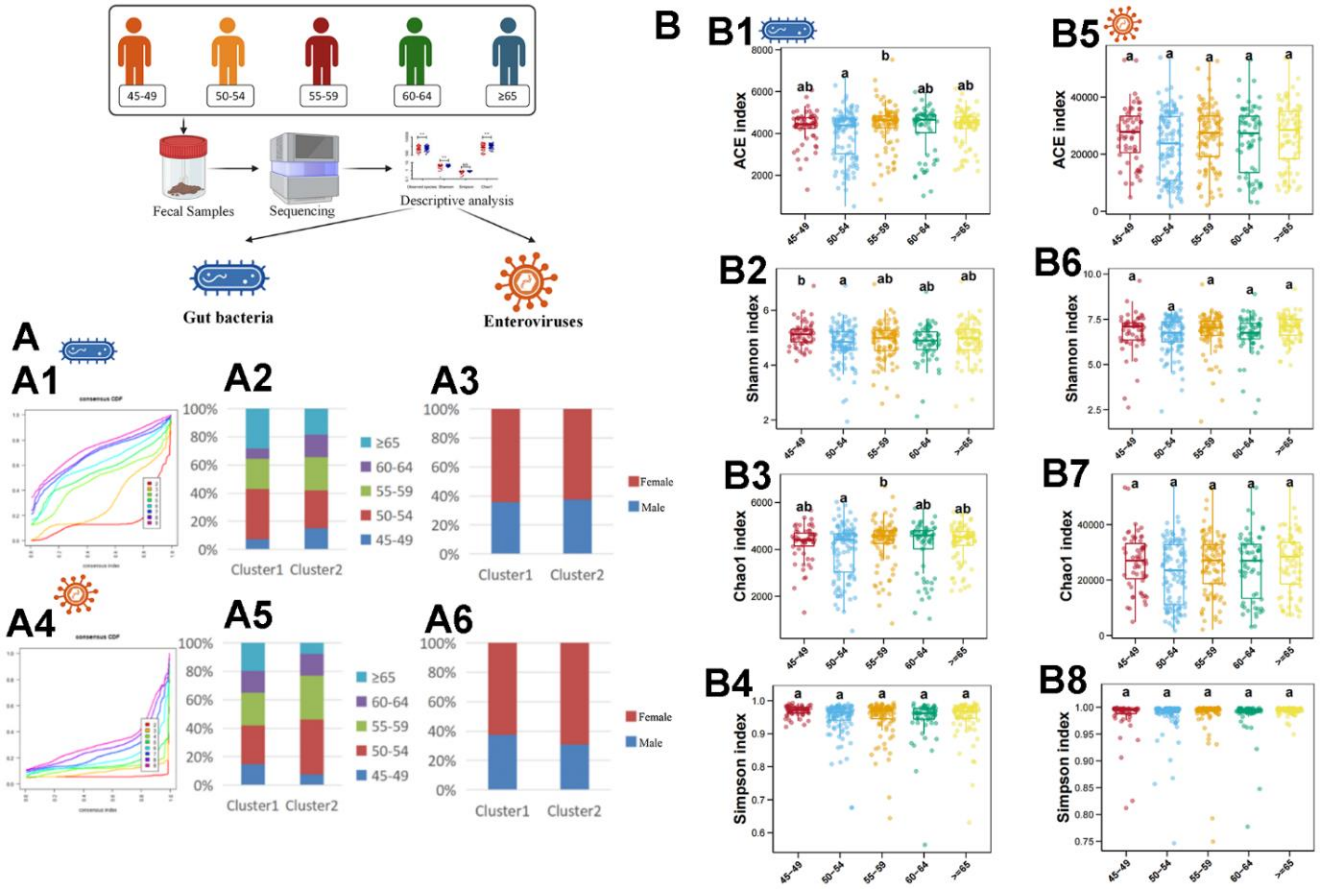
**Descriptive analysis of gut bacteria and enteroviruses.** (**A1**) shows the unsupervised clustering of samples based on gut bacteria in species level. (**A2**) shows the age-related composition ratio of each cluster based on gut bacteria in species level. (**A3**) shows the sex-related composition ratio of each cluster based on gut bacteria in species level. (**A4**) shows the unsupervised clustering of samples based on enterovirus in genus level. (**A5**) shows the age-related composition ratio of each cluster based on enterovirus in genus level. (**A6**) shows the sex-related composition ratio of each cluster based on enterovirus in genus level. (**B1**–**B4**) plots the ACE, Shannon, Chao1, and Simpson index of gut bacteria in species levels, respectively. (**B5**–**B8**) plots the ACE, Shannon, Chao1, and Simpson index of enterovirus in genus level, respectively. The a, b, and ab in (**B**) indicate that there are statistical differences between different labels in different groups.

First, we used an unsupervised clustering method to cluster samples according to the abundance of gut bacteria. The results showed that all samples were divided into 2 clusters at the species level (Figure 1A1). Then, we further analyzed the sex ratio and age composition in different clusters. It was found that the majority of culster1 and culster2 were individuals aged 50-54 (Figure 1A2). Moreover, women predominate in both clusters (Figure 1A3). The similar results were obtained by unsupervised clustering of samples based on abundance of enterovirus (Figure 1A4–1A6). Then, the diversity of different age stratification was analyzed. The results showed that there was no statistical difference in the diversity of the enterovirus at all age groups (Figure 1B5–1B8), while the bacterial diversity was highest in the 45-49 years group (Figure 1B1–1B4).

### Difference analysis based on RF algorithm

**Figure 2:**
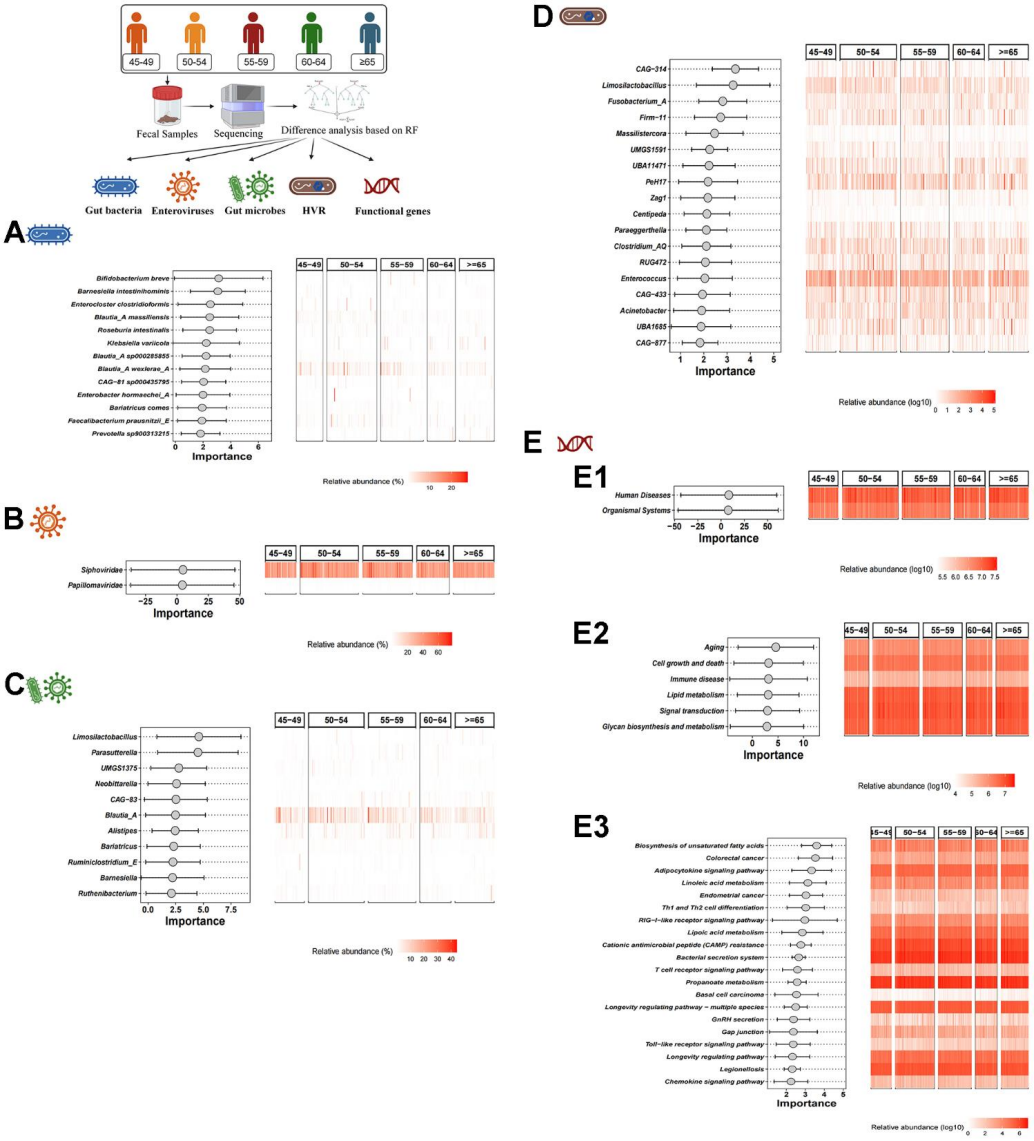
**Difference analysis based on RF algorithm.** (**A**–**D**) show the characteristic gut bacteria, characteristic enteroviruses, characteristic gut microbes and characteristic HVR screened by RF algorithm, respectively. (**E1**–**E3**) show the characteristic functional genes in KEGG level 1, level 2 and level 3 by RF algorithm, respectively.

Based on the RF algorithm, a total of 13 characteristic gut bacteria (including *Bifidobacterium breve*, *Barnesiella intestinihominis* and *Enterocloster clostridioformis*), 2 characteristic enteroviruses (including Siphoviridae and Papillomaviridae), 11 characteristic gut microbes (including *Limosilactobacillus*, *Parasutterella* and *UMGS1375*) and 18 characteristic HVR (including *CAG-314*, *Limosilactobacillus* and *Fusobacterium_A*) were screened in different age groups respectively (Figure 2A–2D). In addition, we also performed KEGG pathway analysis. In KEGG level 1, level 2 and level 3, 2 functional genes (human diseases and organismal systems), 6 functional genes (e.g., aging, cell growth and death, etc.) and 20 functional genes (e.g., Biosynthesis of unsaturated fatty acids, colorectal cancer, etc.) were identified, respectively (Figure 2E1–2E3).

### Difference analysis based on LDA

**Figure 3:**
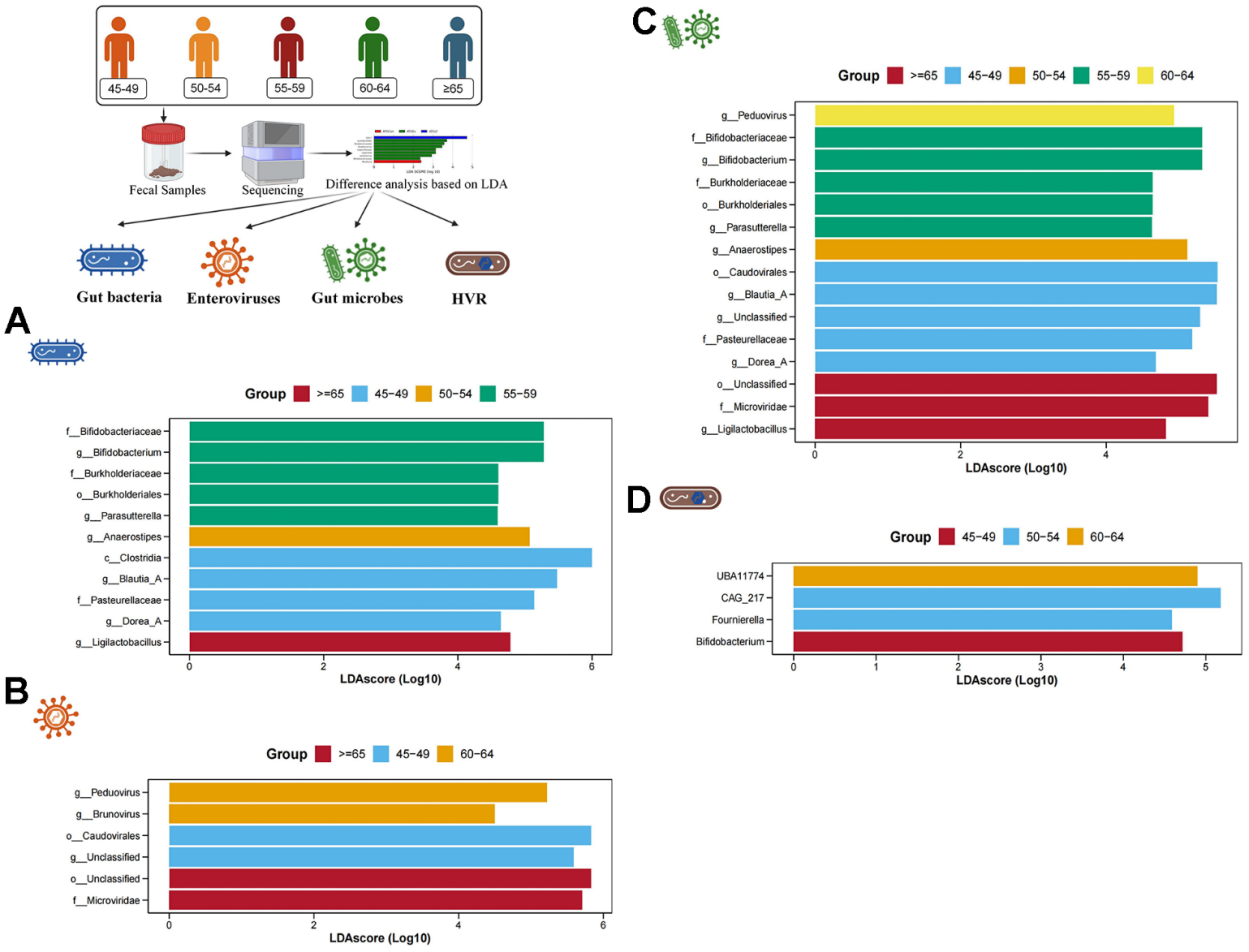
**Difference analysis based on LDA.** (**A**–**D**) show the characteristic gut bacteria, characteristic enteroviruses, characteristic gut microbes and characteristic HVR screened by LDA, respectively. The higher the value of LDAscore, the higher the enrichment degree of the gut microbes in the corresponding group.

Subsequently, based on LDA, we conducted the screening of characteristic gut bacteria, enteroviruses, gut microbes and HVR in different age groups. In the gut bacteria, 5 characteristic bacteria (*Bifidobacterium, Bukholderiales*, *Parasutterell*a, etc.) were enriched in individuals aged 55-59 years, and 4 characteristic bacteria (*Clostridia, Blautia_A*, *Dorea_A* and *Pasteurellaceae*) were enriched in individuals aged 45-49 years. *Anaerostipes* were significantly enriched in individuals aged 50-54, while *Ligiactobacillus* was significantly increased in individuals older than 65 years old (Figure 3A). In the enteroviruses, a total of 6 characteristic enteroviruses were identified in individuals aged 60-64 years (*Peduovirus* and *Brunovirus*), individuals aged 45-49 years (*Caudovirales* and an unclassified virus) and individuals older than 65 years (*Microviridae* and an unclassified virus) (Figure 3B). In the gut microbes, a total of 15 gut microbes have been identified. For example, a characteristic gut microbe (*Peduovirus*) was significantly enriched in individuals aged 60-64 years, 5 characteristic gut microbes (*Bifidobacterium, Burkholderiaceae, Parasutterella,* etc.) were significantly enriched in individuals aged 55-59 years, a characteristic gut microbe (*Anaerostipes*) was significantly enriched in individuals aged 50-54 years, 5 characteristic gut microbes (*Caudovirales, Blautia_A, Dorea_A*, etc.) were significantly enriched in individuals aged 45-49 years, and 3 characteristic gut microbes (*Ligilactobacillus, Microviridae* and an unclassified microbe) were significantly enriched in individuals older than 65 years (Figure 3C). In addition, 4 characteristic HVR were found among 5 different age groups. *UBA11774* was more abundant in individuals aged 60-64, *CAG_217* and *Fournierella* was more abundant in individuals aged 50-54, and *Bifidobacterium* was more abundant in individuals aged 45-49 (Figure 3D).

### Age-related trends in characteristic gut bacteria and enterovirus

**Figure 4:**
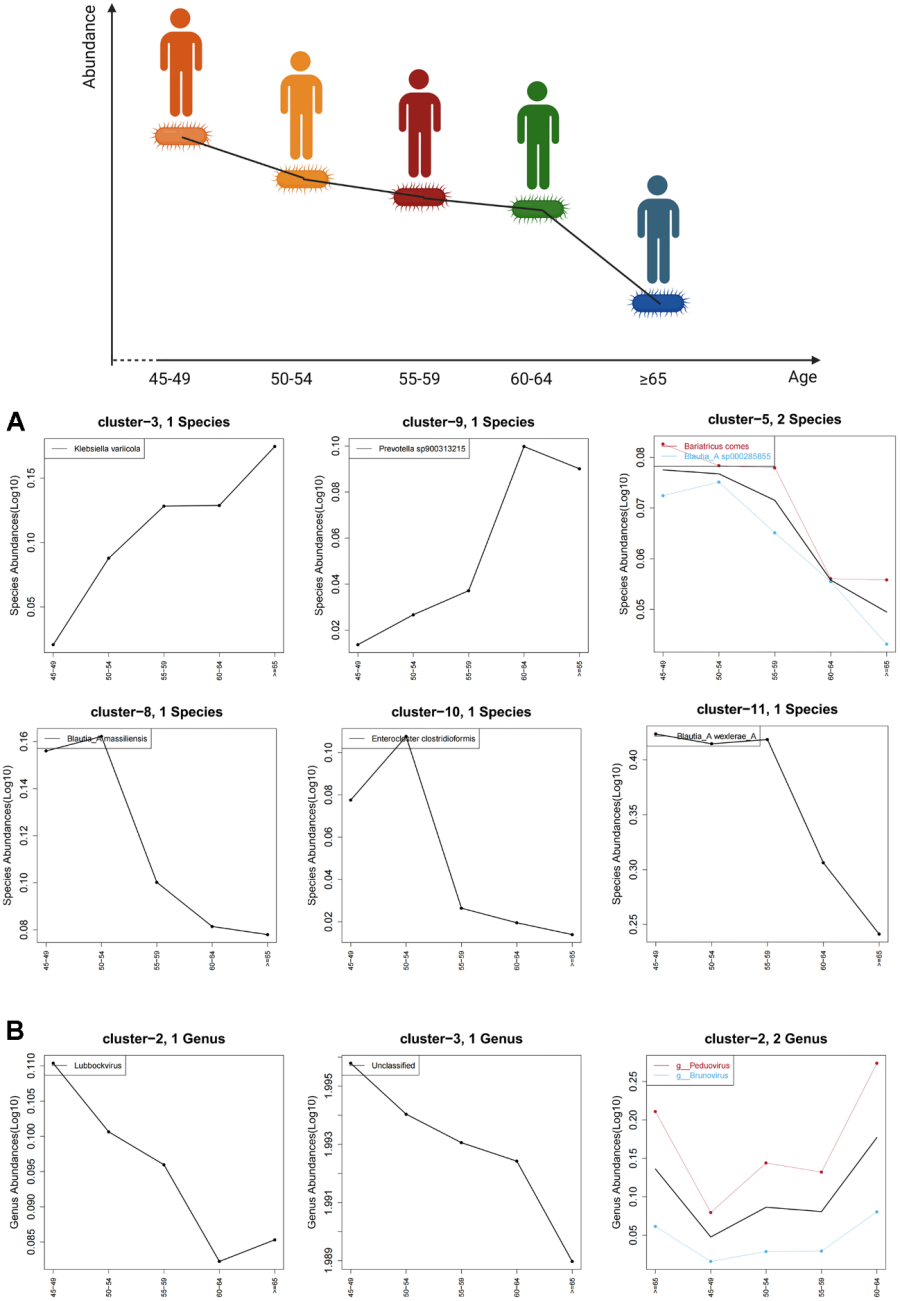
**Age-related trends in characteristic gut bacteria and enterovirus.** (**A**) shows the trend of characteristic gut bacteria with age in species level and (**B**) shows the trend of characteristic enteroviruses with age in genus level. The black curve shows the overall age-related trend of all species in the cluster, while the other colored curves represent age trends for microbes with associated labels.

Age-related trend analysis was performed for all characteristic gut bacteria in species level and all enterovirus in genus level. The abundance of *Blautia_A massiliensis, Blautia_A wexlerae_A* and *Enterocloster clostridioformis* decreased with age, while the abundance of *Klebsiella variicola* and *Prevotella* increased with age (Figure 4A). Moreover, the abundance of *Lubbockvirus* also decreased with age. However, it is difficult to observe a significant age-dependent trend for other enterovirus in individuals older than 45 years (Figure 4B).

## DISCUSSION

This study included a sample of 100 people over the age of 40. The data on the abundance of various gut microbes were obtained through PacBio sequencing. First, characteristic gut bacteria and characteristic enteroviruses in each age group (45-49, 50-54, 55-59, 60-64, ≥65) were found. Then, for these characteristic gut microbes, the trends in their abundance with respect to age were plotted. It was discovered that the abundance of gut microbes such as *Blautia_A massiliensis, Blautia_A wexlerae_A* and *Enterocloster clostridioformis* decreased with age. The abundance of gut microbes, including *Klebsiella variicola, Prevotella* and *Lubbockvirus*, increased with age. At the same time, a joint analysis of gut bacteria and enteroviruses was performed. Finally, it was found that some gut microbes (*Limosilactobacillus, Parasutterella* and *Bifidobacterium, etc.)* were more different among 5 groups.

*Bifidobacterium* that is always considered as probiotics has various biological functions, such as immune regulation [39], anti-tumor activity [40], anti-inflammation [41], and anti-aging activity [42], thereby being significantly associated with human health. Bajic D found that *Bifidobacterium* had an age-dependent difference that would affect the effect of prebiotics. In children, the prebiotic effect was more associated with increased melatonin, while in adults it was associated with a significant increase in folic acid [43]. Since the population for comprehensive physical examination is usually over 40 years old in China, our study focused on the individuals over 40 years old. An age-related difference in *Bifidobacterium* was also found in our study, with *Bifidobacterium* abundance being higher in people aged 55-59 years, which may indicate that other age-prone diseases are associated with fewer *Bifidobacterium*. This may require larger individuals sample collection that is not limited to healthy individuals and further mechanism studies.

*Blautia* that was found to be age-related in this study is an anaerobic bacterium with probiotic characteristics that occur widely in the feces and intestines of mammals [44]. Its probiotic effects include biological transformation and the ability to regulate host health and alleviate metabolic syndrome [45]. A cross-sectional study in Japan, involving the composition of the gut microbiome at all ages, indicated that *Blautia* was more abundant in Japanese adults (21-69 years) [46]. In addition, a study of the long-term monitoring of the human gut microbiota from the 2nd week to 13 years of age found that it was difficult to detect *Blautia* in infants younger than 6 months, but it is common in children over 1-year old [47]. Therefore, it is possible that the overall abundance of *Blautia* first increases and then decreases with age. Our study found that the abundance of *Blautia* decreased with age in individuals over 45 years old. Thus, it may be that the highest abundance of *Blautia* occurs before age 45, but further research is needed to confirm this.

*Enterocloster* is a recently distinguished genus of gut bacteria, including reclassification of 15 taxa [48]. At present, no correlation between *Enterocloster* and age has been reported. We have discovered that the abundance of *Enterocloster clostridioformis* decreased with age for the first time, which may provide a new direction for the study of age and gut bacteria.

Like gut bacteria, it was found that the abundance of many enteroviruses changes with age, such as *Peduovirus, Teseptimavirus* and *Lubbockvirus*. Recent studies have also shown age-dependent changes in human enteroviruses [49], but these analyses were based on data from a public database and did not collect clinical samples for verification. This study more pragmatically found an association between age and enterovirus from clinical samples. Therefore, these results provide new directions for the study on the role of enteroviruses in the physiological and pathological processes of the human body.

Gut bacteria and enterovirus are the main components of gut microbes, and they were analyzed jointly in this study, which showed a more comprehensive view of the characteristic gut microbes. There were some characteristic gut microbes that were not found in the univariate analysis. Considering the complex interaction relationship between gut bacteria and enterovirus [50], we studied from the perspective of HVR. Finally, it was found that 22 characteristic HVR exist in the 5 groups with different ages. *Bifidobacterium* and its infected viruses also had significant differences, and the proportion of infected viruses was the highest in individuals aged 45-49.

Moreover, the differences in functional genes of human diseases revealed the possibility of different onset ages of different diseases. The analysis of functional gene differences proved that age-related changes in gut bacteria were associated with changes in the regulatory pathways of aging, cell growth and death, immune diseases, colorectal cancer, and longevity, which provides molecular target or gene pathway basis for the subsequent research on age-induced diseases or age-related diseases.

However, there are still some flaws in this study. First, the influence of diet, drugs and other factors on gut microbes should not be ignored. More detailed inclusion and exclusion criteria should be developed to further reduce differences in microbiological analysis results due to differences of diet, medication, etc. Then, while characteristic gut microbes at different ages were screened, the role of these gut microbes at different ages and their regulatory mechanisms were unclear. Therefore, further mechanistic studies may be needed in the future. In addition, the specific mechanisms by which these age-related gut microbes cause disease in humans remain unclear. Therefore, further research is needed to further clarify the interactions between gut microbes, age, and disease, and to elucidate the regulatation the mechanisms. Finally, the conclusion of this study is just obtained through bioinformatics analysis. In the future, a series of experiments should be carried out to clarify the axis of age-related changes for gut microbes and provide guidance for human gut microbial health monitoring.

## CONCLUSIONS

In this study, 510 samples were included through bioinformatics analysis to screen out the characteristic gut microbes of individuals in different ages, including gut bacteria, enterovirus and HVR. Most importantly, the abundance of characteristic gut microbes was plotted over time in line charts. Gut microbes in human gut increase or decrease with age. These changes in community structure of gut microbes may be related to age-induced diseases.

## MATERIALS AND METHODS

### Sample collection

The subjects of the study included 510 healthy individuals over 45 years (68 individuals aged 45-49, 136 individuals aged 50-54, 126 individuals aged 55-59, 81 individuals aged 60-64, and 100 individuals older than 65 years old) who were admitted to the health examination center in Huzhou Central Hospital from January 2020 to April 2023. All subjects signed informed consent under the guidelines approved by the Ethics Committee of Huzhou Central Hospital. The information of all subjects was shown in the Table 1.

**Table 1:** The clinical data of samples.

**Group**	**1**	**2**	**3**	**4**	**5**	**p-value**
Age range	45-49	50-54	55-59	60-64	≥65	/
Sample	68	136	125	81	100	/
Male	26	42	50	32	45	0.262
Smoking	30	53	53	30	46	0.722
Drinking	31	70	66	32	59	0.100

Inclusion criteria: (1) individuals with a thorough medical examination; (2) individuals signed a written informed consent form.

Exclusion criteria: (1) patients with serious underlying diseases; (2) patients suffering from mental illness or cognitive and communication dysfunction; (3) patients who received antibiotics and gut bacteria regulation drugs within 3 months.

### Fecal samples

About 5-10 grams of fecal samples from patients who did not use laxatives or lubricants were collected by a fecal collector within half an hour after defecation before breakfast in the morning. The fecal samples were clearly identified and stored in an -80° C ultra-low temperature refrigerator less than 1 month.

Uniform quality control standards for fecal samples: PCR amplification pre-test was used for fecal sample quality control. PCR primers were 27F (AGRGTTYGATYMTGGCTCAG) and 1492R (RGYTACCTTGTTACGACTT). If the band produced by the purified PCR product was too weak, this sample would be removed and a new sample would be included to continue.

### Metagenomic sequencing

Total DNA was extracted from fecal samples using the E.Z.N.A ® fecal DNA kit (Omega Bio-tek, Norcross, GA, USA). The Covaris S220 focused ultrasound instrument (Woburn, MA USA) was used to shear genomic DNA and prepare sequencing libraries with fragments approximately 450 bp in length. The Illumina HiSeq X instrument was used for sequencing in the peer 150bp (PE150) mode. Trimmomatic (http://www.usadellab.org/cms/uploads/supplementary/Trimmomatic) was used to trim the original sequence obtained from the read to remove low-quality data. BWA mem algorithm (http://bio-bwa.sourceforge.net/bwa.shtml) was used to map after quality control data with the human genome. Finally, reads contaminated with host genome and low-quality data were screened out, and other data were used for further analysis. The controlled data were compared with the UHGG database (doi:10.1038/s41587-020-0603-3) and the MGV database (doi:10.1038/s41564-021-00928-6) to identify the bacterial and viral species.

### Taxonomic analysis

Each sequence was annotated for species classification by RDP classifier (https://sourceforge.net/p/rdp-classifier/news/2023/08/rdp-classifier-214-august-2023-released/, version 2.2). Compared with Silva 16S rRNA database (v138), the threshold was set as 80%. To obtain the species classification information corresponding to each OTU, UCLUST algorithm was adopted to conduct taxonomic analysis on the representative sequences of OTU. At each classification level, domain (domain), phylum (phylum), class (class), order (order), family (family), genus (genus) and species (species) were counted for the community composition of each sample. ConsensusClusterPlus was used to analyze different clusters of samples based on the previously obtained gut bacteria in species level and enteroviruses in genus level.

### Diversity analysis

Through Alpha diversity, the study of microbial diversity in community ecology reflects the abundance and diversity of microbial communities, including a series of statistical analysis indexes. This part mainly focuses on Alpha diversity index, including a*bundance-based Coverage Estimator* (ACE), Chao1, Shannon and Simpson.

### Difference analysis

The purpose of random forest (RF) algorithm is to build a model according to the existing data, so as to realize the classification of data and the prediction of other indicators. If the target variable is a categorical variable, the random forest can be classified. If the target variable is continuous, the random forest can perform regression prediction. In the process of building the random forest model, it can also identify potential species that can distinguish the differences between different groups of samples. Moreover, LEfSe can discover high-dimensional biological markers and reveal genomic features. The algorithm emphasized statistical significance and biological relevance and used linear discriminant analysis (LDA) to estimate the magnitude of the influence of each component (species) abundance on the differential effect.

### Kyoto Encyclopedia of Genes and Genomes (KEGG)

The 16s sequencing data were aggregated by closed reference method. Then they were internally standardized, getting normalized species abundance table. Finally, according to the relationship table between genes and KEGG database, expression table of functional genes in three levels was obtained.

### Trend analysis

To study the variation trend of species abundance in different groups, the abundance of all (differential) species obtained according to screening criteria in all group comparisons was analyzed by K-Means clustering. The cluster represents the species with similar trends. Clustering used the KMeans function that comes with R language.

### Availability of data and materials

The datasets generated during the current study can be accessed from the China National GeneBank DataBase (CNGBdb), with the ID of CNP0004360. The corresponding number of samples can be found in Supplementary Table 1.

## Supplementary Material

Supplementary Table 1
